# Empathy moderates the relationship between cognitive load and prosocial behaviour

**DOI:** 10.1038/s41598-023-28098-x

**Published:** 2023-01-16

**Authors:** Roger S. Gamble, Julie D. Henry, Eric J. Vanman

**Affiliations:** School of Psychology, The University of Queensland, Brisbane, Australia

**Keywords:** Psychology, Human behaviour, Public health

## Abstract

Cognitive load reduces both empathy and prosocial behaviour. However, studies demonstrating these effects have induced cognitive load in a temporally limited, artificial manner that fails to capture real-world cognitive load. Drawing from cognitive load theory, we investigated whether naturally occurring cognitive load from the ongoing COVID-19 pandemic moderated the relationship between empathy and prosocial behaviour (operationalised as support for public health measures). This large study in an Australian sample (*N* = 600) identified negative relationships between pandemic fatigue, empathy for people vulnerable to COVID-19, and prosocial behaviour, and a positive relationship between empathy and prosocial behaviour. Additionally, we found that the negative effect of the pandemic on prosocial behaviour depended on empathy for vulnerable others, with pandemic fatigue’s effects lowest for those with the highest empathy. These findings highlight the interrelationships of cognitive load and empathy, and the potential value of eliciting empathy to ease the impact of real-world cognitive load on prosocial behaviour.

## Introduction

Empathy is the ability to understand (cognitive empathy) and experience (affective empathy) the perspective and feelings of another^1–3^ and has been linked to prosocial behaviour in adults and children^4–6^. However, conventional approaches to studying empathy have relied almost exclusively on relating behavioural or neural empathic responses to trait-based indicators^7,8^. Such approaches fail to consider state-level variation in empathy attributable to external factors.

Several recent studies have demonstrated that state empathy reflects a dynamic process, which can be driven by the motivation to avoid engagement for fear of negative consequences or to approach others to obtain socioemotional rewards^9^. Empathy is also influenced by external variables, including physical distance^10^, target race^11^, and group membership^12^. Because empathy is dependent on how we cognitively process these external features, cognitive factors play a crucial role in understanding when and how strongly empathy is elicited.

If cognitive processing is important, then cognitive load should be too. Higher cognitive load decreases performance on various tasks^13^, including social ones. For instance, people experiencing higher cognitive load omit potentially important information when making inferences about others^14^. Finite, top-down working memory resources should also theoretically impose limits on a person’s capacity for empathy. Consistent with this view, increased cognitive load reduces behavioural and neural empathic responses to emotional scenarios^15–18^. Additionally, when attention was directed away from a painful image, neural activity related to the component of the empathic response associated with affect-sharing was unaffected by cognitive load but reduced in the component believed to reflect cognitive evaluation^19^. Cognitive load also appears to influence the downstream effects of empathy. Hiraoka and Nomura asked participants to memorise and retain a two- (low cognitive load) or eight-letter (high load) Latin alphabet string while listening to the sound of a crying infant^20^. Cognitive load decreased caregiving intention and increased intention to neglect the infant, with both these effects mediated by state affective empathy. These reductions in empathy, reduced caregiving intention, and increased neglect intention were also more substantial in the high load than the low load condition, suggesting that cognitive load diminishes prosocial behaviour by depleting cognitive resources. Cognitive load also reduces prosocial behaviour in the form of trust with others^21^, whereas the use of a cognitively effortful emotion regulation strategy (cognitive reappraisal) moderates the relationship between empathy and prosocial behaviour^22^.

However, a critical limitation to these cognitive load studies relates to the nature of the cognitive load manipulation. Typically, cognitive load is manipulated in a temporally limited, artificial fashion—such as by having participants memorise and then retain an alphanumeric sequence or focus on stimuli unrelated to the empathy-inducing stimulus. Perhaps more importantly, cognitive load can persist across extended timeframes in a naturalistic environment because attentional resources are constantly used. Prolonged cognitive load also induces mental fatigue^23^, yet this has not been considered in research on empathy and cognitive load.

The COVID-19 pandemic has provided a unique, powerful, real-world way to test the prediction that cognitive load might influence prosocial behaviour. In response to the pandemic, which has killed millions and infected hundreds of millions more globally^24^, public health measures such as physical distancing^25^, mandated mask-wearing^26^, and lockdowns^27^ have been widely deployed. Although such measures effectively reduced the spread of the virus^28^, they have come at an enormous economic, social, and psychological cost^29^. Consequently, many countries have reported reduced adherence to public health measures owing to mental exhaustion^30^. The World Health Organisation has termed this phenomenon ‘pandemic fatigue’^28^.

In the context of the current study, pandemic fatigue can also be considered a specific, severe form of cognitive load as defined under cognitive load theory. Working memory is the critical mental workspace that allows us to hold and manipulate information, and therefore to reason, learn and comprehend. Central to cognitive load theory is the understanding that because this working memory capacity is limited, greater cognitive load may negatively impact cognitive function and task performance^13,31^. Although cognitive load theory identifies three distinct types of cognitive load, most relevant to understanding the deleterious effects of pandemic fatigue on psychological performance is extraneous load, which refers to unnecessary mental effort that promotes neither task completion or learning^13,31^. Importantly too, empirical studies consistently show how cognitive load exacerbates the effects of stress. For instance, higher stress-induced cortisol levels following extended periods of mental activity compromises performance on tests of executive function^32^, while inducing stress in a high cognitive load condition disrupts attentional processing^33^. Higher levels of cognitive load have also been shown to increase the proclivity to make riskier decisions^34^. Thus, pandemic fatigue can most parsimoniously be explained under the cognitive load theory framework. Specifically, the introduction of new ways of performing routine tasks, the rapidly shifting conditions of the pandemic and the uncertainty about the duration of health measures required additional mental resources to process and contributed to overall stress levels^35^.

Cognitive load theory also provides an elegant explanation of why those who are high in information fatigue choose to reduce their knowledge of COVID information: they engage in avoidant behaviour precisely *because* it is so prevalent. Avoidance of COVID-19 information has been linked to perceived information overload in a German setting^36^, and information avoidance predicted reluctance to engage in COVID public health measures in a Chinese setting^37^. Thus, the accumulation of novel tasks and environmental stressors during the pandemic introduce extraneous cognitive load that depletes working memory capacity, which negatively impacts broader cognitive function and behaviour,

Importantly, adherence to public health measures should be linked to empathy because it is a form of prosocial behaviour^38–40^. This is because perceptions of civic duty and benefits to others represent prosocial motivations, and empirical data supports this view. For instance, a recent study using cross-sectional research methods showed that stronger prosocial motivations in general were associated with increased adherence to physical distancing^41^. Further, these effects have also been demonstrated using both longitudinal and experimental research methods. Using group-based trajectory modelling to track adherence to physical distancing over an 8 month period, one of the most important predictors of poorer adherence trajectories was a lower prosocial attitude towards physical distancing^42^. Experimental studies have also shown that when physical distancing is framed as a prosocial behaviour, adherence behaviour is subsequently greater relative to messaging that uses either self-interested or threatening language^43,44^.

Empathy for people vulnerable to COVID-19 has also been directly linked to greater adherence to physical distancing and mask-wearing, and inducing empathy promoted greater adherence to these health measures^45^. In addition, empathy uniquely predicted acceptance of disruptive mandates (e.g., lockdowns) as effective in reducing virus spread; distress did not influence this relationship^46^. Moreover, higher state empathy and experimental inducement of empathy each result in increased intention to vaccinate oneself against COVID-19^47^, with COVID-19 vaccination status also predicted by prosociality relating to protecting others from disease^48^. Such evidence supports the view that empathy and adherence to COVID-19 health measures are related.

As noted previously, because pandemic fatigue is a specific type of cognitive load, it provides a powerful way to test the real-life impact of cognitive load on empathy and prosocial behaviour. When this study was conducted, Australia was ideal for testing this hypothesis because widespread vaccination had not been achieved^49^, and repeated, prolonged city-wide and state-wide lockdowns were common^50,51^. Moreover, the extension of measures such as mandatory mask-wearing, enforcement of physical distancing, and limits on gatherings became features of life in pandemic-era Australia (additional context for Australia during the COVID-19 pandemic is in Supplementary Materials)^52^.

In American and French samples, it has been shown that compliance with public health measures can be impacted by individual differences in affective dispositions, such that moral values emphasizing care and pathogen disgust sensitivity increase public health compliance, and affective pushback against threats to personal liberty reduce compliance^53^. Based on this, the Australian experience of the COVID-19 pandemic and the real limitations on personal freedom due to the imposition of unpredictable and severe measures might have predicted heightened, widespread resistance from the population regarding public health measures. However, this was not generally seen and likely reflects that Australians place less emphasis on personal liberty relative to their American or British counterparts. During the pandemic, most Australians were willing to comply with prolonged personal restrictions in the interest of collective protection against the virus^54–56^. Therefore, pandemic fatigue in the Australian context more strongly reflects a measure of exhaustion with ongoing pandemic information and restrictions, and not a reaction to infringements on personal liberty.

In light of such chronic and extreme levels of pandemic fatigue among Australian residents^57,58^, this country provided a perfect location to test the central research question of whether cognitive load influences the relationship between empathy and prosocial behaviour (operationalised here as support for public health measures) in a real-world setting. The central hypothesis was that pandemic fatigue would decrease support for public health measures but that empathy would moderate this relationship with higher empathy buffering the negative impact of pandemic fatigue on motivation to engage in public health measures.

This study tested whether empathy was related to adherence to health measures and vaccination intention in a pandemic-fatigued population. Specifically, we hypothesised a positive relationship between empathy, support for public health measures, and vaccination intention. We also predicted a negative relationship between pandemic fatigue and empathy, support for public health measures, and vaccination intention. In addition, we assessed whether increasing empathy for people vulnerable to COVID-19 influences motivation to adhere to health measures and vaccination intention in a pandemic-fatigued population. We, therefore, hypothesised that empathy would moderate the effects of pandemic fatigue to increase support for public health measures and vaccination likelihood.

## Results

To test the first hypothesis that there would be a positive relationship between empathy, support for public health measures, and vaccination intention, and a negative relationship between pandemic fatigue and empathy, support for public health measures, and vaccination intention, we first conducted a series of correlation analyses (see Table 1). There was a strong positive relationship between empathy and support for public health measures (*r*[598] = 0.515, *p* < 0.001), a weak positive relationship between empathy and vaccination status (*r*[598] = 0.128, *p* = 0.002), and a weak positive association between vaccination status and support for public health measures (*r*[598] = 0.172, *p* < 0.001). A weak negative relationship was identified between pandemic fatigue and empathy (*r*[598] = − 0.204, *p* < 0.001), with a moderate negative relationship between pandemic fatigue and support for public health measures (*r*[598] = − 0.384, *p* < 0.001) and a weak negative association between pandemic fatigue and vaccination status (*r*[598] = − 0.110, *p* = 0.007). Location information was used to create a lockdown status index (lockdown and no lockdown). Lockdown status was considered as an alternative predictor of pandemic fatigue because public health orders are the most restrictive during periods of lockdown (and so pandemic fatigue would be expected to be higher). This also revealed a weak positive correlation between lockdown status and empathy (*r*[598] = 0.089, *p* = 0.029), a weak positive association between lockdown status and support for public health measures (*r*[598] = 0.123, *p* = 0.002), a weak positive relationship between lockdown status and pandemic fatigue (*r*[598] = 0.252, *p* < 0.001), and a weak positive association between lockdown status and vaccination status (*r*[598] = 0.155, *p* < 0.001). Correlation analyses were conducted for the Study 1 dataset (Table S1).

**Table 1:** Correlation matrix of age, empathy, support for public health measures, pandemic fatigue, vaccination status (yes/no), vaccination willingness and lockdown status (no lockdown, lockdown). Pearson’s *r*, with 95% confidence intervals shown in brackets.

	Age	Empathy	Public health	Pandemic fatigue	Vaccination status	Vaccination willingness	Lockdown
Age	–						
Empathy	− 0.044	–					
	[− .123, .036]						
Public health	− .148***	.515***	–				
	[− .225, − .126]	[.453, .571]					
Pandemic fatigue	− 0.054	− .204***	− .384***	–			
	[− .133, .027]	[− .28, − .126]	[− .45, − .314]				
Vaccination status^1^	0.046	.128**	.172***	− .11**	–		
	[− .034, .126]	[.048, .206]	[.093, .248]	[− .189, − .03]			
Vaccination willingness	− 0.044	.106**	.201**	− 0.066	− .10*	–	
	[− .124, .036]	[.026, .185]	[.123, .277]	[− .146, .014]	[− .179, − .02]		
Lockdown^a^	− .155***	.089*	.123**	.252***	.155***	0.005	–
	[− .232, − .076]	[.009, .168]	[.043, .201]	[.176, .326]	[.075, .232]	[− .075, .085]	

^a^ = point biserial correlation was calculated.

*p < .05, **p < .01, ***p < .001.

To test the second hypothesis that empathy would moderate the effect of pandemic fatigue on support for public health measures and vaccination intention, we ran separate hierarchical multiple regressions for each outcome variable. A moderated hierarchical linear regression investigating support for public health measures as the outcome variable was conducted by assessing pandemic fatigue, empathy, and empathy × pandemic fatigue interaction as predictors (see Table 2). Model 1 incorporating pandemic fatigue explained 14.5% of the total model variance, with pandemic fatigue decreasing support for public health measures (*p* < 0.001). Including empathy in the second model explained an additional 19.7% of the model variance (*p* < 0.001), with pandemic fatigue reducing and empathy increasing support for public health measures (*p*’s < 0.001). Model 3, which included the empathy × pandemic fatigue interaction, accounted for an additional 3.3% of the variance, with pandemic fatigue reducing support for public health measures (*p* < 0.001), empathy × pandemic fatigue increasing support for public health measures (*p* < 0.001), and empathy no longer contributing to support for public health measures (*p* = 0.777).

**Table 2:** Hierarchical linear regression of support for public health measures predicted by pandemic fatigue, empathy, and empathy × pandemic fatigue interaction.

	β	95% Confidence Interval		*t*	*p*	Δ*R*^*2*^	AIC
Variable		Lower	Upper				
Block 1						.145	1202.558
Intercept				64.779	< .001***		
PF	− .382	[− .457,	− .308]	− 10.088	< .001***		
Block 2						.197	1047.591
Intercept				16.354	< .001***		
PF	− .292	[− .359,	− .226]	− 8.62	< .001***		
Empathy	.453	[.387,	.52]	13.365	< .001***		
Block 3						.033	1019.002
Intercept				12.006	< .001***		
PF	− .284	[− .349,	− .219]	− 7.13	< .001***		
Empathy	.405	[.338,	.472]	− 0.284	.777		
Empathy × PF	.16	[.104,	.216]	5.584	< .001***		

*PF* pandemic fatigue, *AIC* akaike information criterion.

*p < .05, **p < .01, ***p < .001.

**Figure 1:**
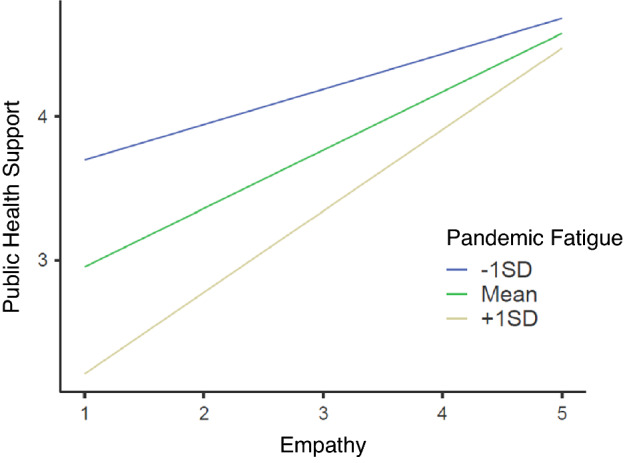
Estimated marginal means graph of state empathy plotted against support for public health measures, divided by low (− 1SD), average and high (+ 1SD) pandemic fatigue in Study 2. When empathy was high, there was no significance difference of pandemic fatigue on support for public health measures. However, when empathy was low, increasing pandemic fatigue decreased support for public health measures and decreasing pandemic fatigue increased support for public health measures.

Comparing the models’ AICs indicated that Model 3 was the best predictive model. Estimated marginal means analysis indicated that state empathy moderated the pandemic fatigue effect on support for public health measures, such that support for public health measures was stronger in high-empathy participants regardless of pandemic fatigue and support for public health measures was weaker in low-empathy participants who had high pandemic fatigue compared to low-empathy participants with low pandemic fatigue (see Fig. 1). A hierarchical binomial logistic regression was then conducted to determine the sequential effect of pandemic fatigue, state empathy and empathy × pandemic fatigue interaction on the likelihood that participants were vaccinated against COVID-19 (see Table 3). Model 1 incorporating pandemic fatigue was significant (*p* = 0.007), accounting for 1.9% of the total variance, with pandemic fatigue decreasing the likelihood of being vaccinated (*p* = 0.007). In Model 2, including empathy was significant (*p* = 0.001), accounting for 3.4% of the total variance, with empathy increasing (*p* = 0.035) and pandemic fatigue decreasing (*p* = 0.011) vaccination probability. Model 3 incorporating empathy × pandemic fatigue was not significantly different from Model 2 (*p* = 0.557). Assessing the AICs showed that Model 2, which included state empathy and pandemic fatigue separately, was the most predictive model.

**Table 3:** Hierarchical binomial logistic regression of vaccination status predicted by pandemic fatigue, empathy, and empathy × pandemic fatigue interaction.

Variables	OR	95% Confidence Interval		*Z*	*p*	Δ*R*_*N*_^*2*^ (χ^2^_Sig_)	AIC
		Lower	Upper				
Block 1						.019 (.007**)	631.501
Intercept				6.643	< .001***		
PF	0.829	[0.724,	0.951]	− 2.682	.007**		
Block 2						.015 (.012*)	627.188
Intercept				0.598	.55		
PF	0.861	[0.748,	0.989]	− 2.11	.035*		
Empathy	1.395	[1.079,	1.803]	2.543	.011*		
Block 3						.001 (.557)	628.842
Intercept				− 0.291	.771		
PF	1.049	[0.534,	2.06]	0.14	.889		
Empathy	1.719	[0.819,	3.608]	1.433	.152		
Empathy × PF	0.954	[0.816,	1.116]	− .588	.557		

The outcome variable is the probability of being vaccinated against COVID-19.

*OR* odds ratio, *PF* pandemic fatigue, *AIC* akaike information criterion, *R*_*N*_^2^ nagelkerke’s *R*^2^.

*p < .05, **p < .01, *** *p* < .001.

Finally, exploratory analyses investigating the effect of vaccination status on state empathy, support for public health measures, and pandemic fatigue using three two-tailed Mann–Whitney *U* tests while using the Benjamini–Hochberg procedure^59^ to control the false discovery rate (FDR) revealed significant effects of vaccination status on state empathy (Yes: *M* = 4.43, *Mdn* = 4.667, *SD* = 0.696; No: *M* = 4.211, *Mdn* = 4.333, *SD* = 0.754; *U*[*N*_Yes_ = 467, *N*_No_ = 133] = 24,694.50, *p* < 0.001, *r*_rb_ = 0.205), support for public health measures (Yes: *M* = 4.37, *Mdn* = 4.5, *SD* = 0.668; No: *M* = 4.075, *Mdn* = 4.333, *SD* = 0.819; *U*[*N*_Yes_ = 467, *N*_No_ = 133] = 23,693.50, *p* < 0.001, *r*_rb_ = 0.237), and pandemic fatigue (Yes: *M* = 3.782, *Mdn* = 3.833, *SD* = 1.437; No: *M* = 4.162, *Mdn* = 4.167, *SD* = 1.385; *U*[*N*_Yes_ = 467, *N*_No_ = 133] = 26,442.0, *p* = 0.009, *r*_rb_ = 0.149). These analyses indicated that vaccinated participants had higher empathy for COVID-vulnerable others, were more supportive of public health measures and experienced less pandemic fatigue than unvaccinated participants.

## Discussion

This study provided partial support for our hypotheses. In support of our first hypothesis, pandemic fatigue was negatively associated with empathy, support for public health measures, and vaccination status. Additionally, consistent with prior research^45,46^, there was a strong positive correlation between empathy and support for public health measures. The presence of lockdowns was also associated with increased pandemic fatigue, empathy for COVID-vulnerable others, support for public health measures, and an increased likelihood of being vaccinated. In support of our second hypothesis, state empathy moderated the relationship between pandemic fatigue and support for public health measures by lessening the negative impact of pandemic fatigue on support for public health measures while maintaining this relationship in low-empathy participants. For vaccination status, empathy and pandemic fatigue contributed to the likelihood of being vaccinated, with the interaction of empathy × pandemic fatigue not affecting the probability of being vaccinated. These results provided partial support for our second hypothesis.

Of note in this study was the finding that state affective empathy moderated the relationship between naturalistic cognitive load and prosocial behaviour. Specifically, although cognitive load due to pandemic fatigue reduced prosocial behaviour, higher state empathy mitigated this detrimental effect of cognitive load on prosocial behaviour, with cognitive load having its most severe impact on prosocial behaviour in those with lower empathy. However, this study also revealed that specific prosocial behaviours like vaccination likelihood were influenced by pandemic fatigue and state empathy, suggesting that empathy and cognitive load contributed to specific prosocial action.

Prior research assessing adherence to COVID-19 public health measures have noted a range of factors that include but are not limited to point to trust in government, interpersonal trust, autonomous motivation, political ideology in the United States, social dominance orientation, trust in science, and psychological reactance^53,60–64^. Together, these highlight the complexity in attributing support for public health measures solely to prosocial intent or behaviour. However, our finding that empathy modulated support for prosocial behaviour is consistent with other findings on empathy and prosocial behaviour during the COVID-19 pandemic^45,47^. These data are also compatible with the altruism hypothesis of empathy, in which a central tenet is that empathy, specifically empathic concern for others, plays a causal role in altruistic motivation that then influences prosocial behaviour^4^. Importantly, this hypothesis identifies two paths whereby cognitive processing may influence prosocial behaviour, namely during the initial processing of affective empathy and evaluating the necessity of altruistic responding^65^. Given the central role of cognitive processing in this model, empathy may be susceptible to interference from cognitive load.

As revealed in our best-fitting model, state empathy positively predicted, and cognitive load negatively predicted, vaccination likelihood, the former finding similar to Pfattheicher^47^. Past research has also revealed that prosociality increased the intention to vaccinate against COVID-19, whereas religiosity and belief in conspiracy theories reduced vaccination intention^48^. The current results combined with prior literature highlight the importance of considering empathy, prosocial behaviour measures, and individual difference characteristics when investigating prosocial action contributors.

At the same time, our study extends the literature on cognitive load interactions with empathic processing in several meaningful ways. It is the first to assess the relationship between empathy and prosocial behaviour when subjected to a *naturalistic* cognitive load. Because we successfully replicated findings from previous studies investigating the effect of cognitive load on empathy^16–19^ and prosocial behaviour^20^, we showed that cognitive load could be induced from real-world circumstances. Our findings also align with recent research into cognitive load effects on empathy. For instance, Bajouk found that cognitive load selectively reduced empathy for pain, negative emotions, and positive emotions in people with low trait empathy^15^.

However, another study revealed that, when listening to a crying infant, people under cognitive load were more likely to neglect a crying infant if they had high trait affective empathy than those with low trait affective empathy^66^. This latter finding appears inconsistent with our own that high affective empathy negated the effect of cognitive load on prosocial behaviour compared to low empathy but may reflect a function of the “online cognitive load” paradigm used to induce cognitive load in Hiraoka^66^, which required participants to memorise an alphanumeric sequence while presented with a target. This is very different from the approach taken in our study, which used an “offline cognitive load” design, where the empathic task was conducted after the cognitive load task^67^. It seems likely that the method used here may have produced weaker cognitive load effects than an online cognitive load paradigm. Thus, using an online design to investigate the moderating effect of trait empathy on the relationship between cognitive load and prosocial behaviour would be a valuable approach to test this possibility directly.

In addition to the theoretical value, our results also have methodological implications for cognitive load research. They suggest that working memory resources in day-to-day life may carry over into experimental settings. Participants in past cognitive load research were assumed to begin from a non-cognitively loaded baseline, which had not accounted for individual variations in working memory capacity. Researchers in future cognitive load studies should attend more closely to whether participants are cognitively loaded at baseline or, if not possible, monitor individual cognitive load before the study begins.

The findings from this study also highlight the importance of understanding cognitive processing in conjunction with empathy. A recent neuroimaging meta-analysis suggested that affective empathy is one component of the hierarchical multicomponent construct of social cognition and identified an intermediate state that co-activated neural regions involved in affective and cognitive empathy^68^. These findings are consistent with a model of empathy that conceptualises the affective and cognitive components of empathy not as a dichotomy but as an overlapping spectrum in which affect-sharing with others is modulated by cognitive processing, and perspective-taking is influenced by empathic concern^69^. Indeed, there is a causal link between metacognitive ability such as self-concept clarity and affective empathy that influences helping behaviour^70^, thus underlining the necessity of higher-order processes in emotional empathy.

It should be noted that our study examined empathy specifically toward people who were vulnerable to COVID-19. Although most empathy research focuses on observer traits, it is important to emphasise that empathy is an interpersonal process between an observer and a target, and features of the target may meaningfully impact on empathy that an observer feels for a target^71,72^. For example, targets who are perceived as vulnerable on the basis of physical features or socioeconomic status elicit more empathy from observers than targets with more similarity to the observer^73–75^. If empathy in our study was defined alternatively to focus on a target from a different group (such as those most affected economically or socially by COVID public health measures), this may have impacted our results and interpretation. Thus, the way empathy is framed is an important consideration in the interpretation of our findings that empathy for COVID-vulnerable people moderates the relationship between cognitive load and prosocial behaviour.

Finally, it is worth noting that after the data were collected for this study, Hajek reported the results of a similar COVID-19 project that examined demographic contributors to trait empathy and trait altruism^76^. The results revealed that trait altruism, but not trait empathy, was associated with vaccination against COVID-19. Although these findings are important in understanding factors contributing to vaccination likelihood, a key difference between the two studies is that our study assessed *state* empathy as a predictor variable and specific prosocial behaviours in the form of support for public health measures and vaccination against COVID-19 as outcome variables. Recent research has revealed an important divergence between state and behavioural indicators of empathy^77^, which are often only negligibly correlated.

Some limitations of this study need to be acknowledged. Firstly, owing to its cross-sectional nature, it is not possible to draw conclusions about potential causality. While it would be valuable to re-conduct this as an experimental study to assess the causality of these findings, given the unique context of the study situated in the pandemic which included city- and state-wide lockdowns it may be extremely difficult to replicate experimentally. Secondly, single items measures were used to index the constructs of mask-wearing and physical distancing here. Thirdly, some of public health questions may not have been worded appropriately for the context. For example, the mask-wearing item from Pfattheicher^45^ asked how much participants “will” wear a mask, which was originally measured when mask-wearing was voluntary. Thus, our responses may have reflected mask-related mandates across jurisdictions. Although we made a composite public health measure to ensure that no individual item was given too much weight, future studies should update public health questions to be contextually relevant. Second, we did not measure some potentially relevant individual difference characteristics. For example, the effects of trait empathy may depend on cognitive load too^15,66^, and other individual- and group-level differences including but not limited to political belief, community identification, employment status and conspiracist belief may have accounted for changes in state empathy, support for public health measures or vaccination^39,48,61,76,78^. Indeed, a cross-sectional psychometric analysis of the Pandemic Fatigue Scale found that pandemic fatigue was highest among those who believed they were unlikely to be infected, believed the disease was mild and had low concern about the pandemic overall^79^. Thus, it is possible that our constructs are affected by the third variable of scepticism to the disease and the pandemic, which would include belief in COVID misinformation.

Finally, we only assumed here cognitive load in the context of the pandemic. Although we found that pandemic fatigue reflected cognitive load, cognitive load itself was not directly induced or measured. We deliberately tried to capture naturally occurring cognitive load, and prior laboratory research supported our predictions that pandemic fatigue would introduce substantial and chronic cognitive load in the specific cohort being assessed. However, direct manipulation of cognitive load in future studies would provide stronger evidence for our model. One way in which future studies might directly manipulate cognitive load would be by presenting participants with pandemic information they should memorise or asking people how much they think about the pandemic in daily life.

It is also important to acknowledge that our findings were from a single Western country during a global pandemic. Assessments of psychological literature have highlighted the disproportionate use of American and other White-Western populations in psychological research despite their statistical infrequency relative to the global population, and data taken from Western populations may not generalise to the human population at large^80–83^. Therefore, it would be critical for behavioural science to incorporate representative and cross-cultural samples from non-Western countries to determine if the model of cognitive load, empathy, and prosocial behaviour proposed here can be cross-culturally validated.

## Conclusion

In conclusion, we used a paradigm of naturalistic cognitive load to assess the impact of empathy on the relationship between cognitive load and prosocial behaviour in the COVID-19 pandemic. While cognitive load in the form of pandemic fatigue decreased prosocial behaviour by inducing mental fatigue, empathy moderated the effect of cognitive load on prosocial behaviour and specific prosocial action by reinvigorating motivation to protect others that, in turn increased prosocial behaviour. However, we did not find a direct effect of empathy manipulation on prosocial behaviour intention. These findings highlight those real-world circumstances can influence the association between empathy and prosocial behaviour, and secondarily in understanding factors that contribute to or detract from support for health measures that may be used to ensure robust public health responses.

## Methods

### Participants

Australian participants were recruited via Prolific in September 2021. They completed a short survey regarding their “attitudes about the coronavirus (COVID-19) pandemic”. Participants were 18 or over and resided in Australia before March 20, 2020, when the international border was closed. A power analysis determined that at least 202 participants would provide 95% power to detect an effect of *r* > 0.25 at *p* < 0.05 using a two-tailed test, and 302 participants would provide 95% power to detect an effect of Cohen’s *f* > 0.25 at *p* < 0.05 for a multiple linear regression^84^. Data were collected from 604 participants, with four excluded from data analysis for not meeting the residency criterion, resulting in a sample of 600 participants (356 female, 232 male, 9 non-binary, 3 N/A; *M* = 29.6 years, *SD* = 12.5; 467 vaccinated, 133 unvaccinated; additional demographic factors in Supplementary Materials). All participants passed the attention check.

All participants provided informed consent to participate in the experiment, which received approval from the Human Research Ethics Committee at the University of Queensland. The study was conducted in accordance with the Declaration of Helsinki. All participants were financially compensated for study participation.

### Study materials and procedure

Participants rated their empathy for people most vulnerable to COVID-19 taken from Pfattheicher^45^. This measure consisted of three items adapted from the Empathic Concern subscale of the Interpersonal Reactivity Index, such as “I feel compassion for those most vulnerable to COVID-19” (see Supplementary Materials)^8^. These three scores were averaged to generate a final empathy score, with Cronbach’s alpha = 0.876.

They also reported the extent they supported public health measures taken to control COVID-19^45,46^. These included one item about physical distancing, one item about mask-wearing, and four items about lockdowns (e.g., “Lockdowns are very effective in stopping the spread of COVID-19”; Cronbach’s α = 0.846). For all the items mentioned above, participants used a 5-point Likert scale (1 = *strongly disagree*, *5* = *strongly agree*). The final score of support for public health measures was created through a mean of all the items (Cronbach’s α = 0.806).

Participants indicated whether they had received at least one dose of a COVID-19 vaccine. Those who selected “yes” were asked how soon they received a vaccine when made available to them on a 4-point scale ranging from 1 (“3+ months after it was made available”) to 4 (“As soon as it was made available”). Those who selected “no” reported whether they intend to get vaccinated on a 5-point scale used by Pfattheicher^47^, ranging from 1 (“I will definitely *not* get vaccinated”) to 5 (“I will definitely get vaccinated”).

Participants then completed the Pandemic Fatigue Scale, asking them to rate six items about pandemic fatigue on a 7-point scale ranging from *strongly disagree* to *strongly agree*^85^. Pandemic fatigue in this scale was divided into the two factors of information fatigue (e.g. “I am sick of hearing about COVID-19”) and behavioural fatigue (e.g., “I am losing my spirit to fight against COVID-19”). The six items were averaged to create a final score for pandemic fatigue (Cronbach’s α = 0.866), with the items for information fatigue and behavioural fatigue averaged to create separate scores for information fatigue (Cronbach’s α = 0.871) and behavioural fatigue (Cronbach’s α = 0.781). Finally, they completed demographic questions relating to their gender, age, nationality, the country they lived in the longest, current location, and a screening question about Australian residency. Finally, participants completed a brief attention check item. Data files and study materials are available at https://osf.io/c7hra/?view_only=9074096e5f73458eba1aa36835e62453.

### Statistical analysis and pre-registration

Statistical analyses were performed on jamovi version 2.2.5.0^86^. The structural validity of each scale was tested using Reliability Analysis (Cronbach’s alpha). The alpha level set to determine significance for this study was 0.05, with the FDR for multiple comparisons controlled to α_FDR_ = 0.05 using the Benjamini–Hochberg procedure. 95% confidence intervals are provided for *r* values in the Pearson correlation analysis, standardised beta coefficients in the multiple linear regression analysis, and odds ratios in the binomial logistic regression analysis. Model fit for individual models in the multiple regression analyses were measured using the Akaike information criterion (AIC). The normality distribution of the variables was assessed using a series of Shapiro–Wilk tests. The tests for state empathy, pandemic fatigue, support for public health measures and vaccination willingness were significant (*p*’s < 0.001), indicating that the distribution for all the variables violated the assumption of normality. Accordingly, non-parametric tests were conducted for all statistical analyses.

In pre-registration, this project was designed as two separate studies involving correlational analysis as Study 1 (https://osf.io/vxyhr/?view_only=433432c34f5441e49451d3abe0265ecf) and an experimental study including an empathy manipulation as Study 2 (https://osf.io/3buzr/?view_only=e7450f62ce524fbdb05d6eb294c8d390). Pre-registration for Study 1 was completed after data collection had taken place, but Study 2 was pre-registered before data collection. However, statistical analyses were not conducted on any of the data until after pre-registration. Thus, not all this study’s data collection was pre-registered but the study predictions and analyses were pre-registered. The studies used independent participants and the same questionnaire measures. However, the empathy manipulation in Study 2 was not significant for state empathy, support for public health measures or vaccination intention among those who hadn’t received a COVID-19 vaccine on three two-tailed Mann–Whitney *U* test with the Benjamini–Hochberg procedure applied to control the FDR (*p*’s > 0.042), we deviated from the pre-registered designs to combine the datasets for statistical analyses. We chose to use the more proximal measure of state empathy as a predictor variable in subsequent analyses. Datasets for Study 1 and 2 were analysed separately prior to dataset combination with results in Supplementary Materials (Tables S1–S3), which found similar results to the analyses conducted using the combined dataset.

## Supplementary Information

Supplementary Information.

## Data Availability

The data that support the findings of this study are openly available in the Open Science Framework at https://osf.io/c7hra/?view_only=9e42dd97813b4725be9a839a22ea9c71.
