# Supplementary material for: Empathy moderates the relationship between cognitive load and prosocial behaviour
Source: Sci Rep. 2023 Jan 16;13:824. doi: 10.1038/s41598-023-28098-x (PMC9841498; doi:10.1038/s41598-023-28098-x)
Supplement: Supplementary file 1 — Supplementary Information. [file 41598_2023_28098_MOESM1_ESM.docx]

**Empathy moderates the relationship between cognitive load and prosocial behaviour – Supplementary Materials**

**Roger S. Gamble**^1,*^, **Julie D. Henry**^1^ **and Eric J. Vanman**^1^

^1^School of Psychology, The University of Queensland, Brisbane, Australia

^*^Corresponding author email: [roger.gamble@uq.net.au](mailto:roger.gamble@uq.net.au)

Table of Contents

Study Items…...………………………………………………………………………………..3

Miscellaneous methods………………………………………………………………………..9

Sample characteristics………………………………………………………………………..10

Texts used in Study 2………………………………………………………………….……..11

Study 1 and 2 analyses.………………………………………………………………………13

Exploratory analyses. ………………………………………………………………………..18

COVID-19 pandemic in Australia……………………………………………………………24

Supplementary materials references………………………………………………………….25

Study Items

*Measures are displayed in order of appearance, but individual item order within each measure was randomised.*

Empathy questions

“The following statements inquire about those vulnerable to COVID-19. Please read each statement and select the response that applies to you most accurately. There are no right or wrong answers. Do not spend too much time on any statement and answer as honestly as you can.”

| I am very concerned about those most vulnerable to COVID-19 | 1 (strongly disagree)  -  5 (strongly agree) |
| --- | --- |
| I feel compassion for those most vulnerable to COVID-19 | 1 (strongly disagree)  -  5 (strongly agree) |
| I am quite moved by what can happen to those most vulnerable to COVID-19 | 1 (strongly disagree)  -  5 (strongly agree) |

Public health measure questions

“The following statements inquire about your thoughts on health measures to curb COVID-19. Please read each statement and select the response that applies to you most accurately. There are no right or wrong answers. Do not spend too much time on any statement and answer as honestly as you can.”

*Physical distancing (“social distancing”)*

| Because of COVID-19, I am massively curtailing my social contact (so-called "social distancing") | 1 (strongly disagree)  -  5 (strongly agree) |
| --- | --- |

*Mask-wearing*

| During the coming days, I will wear a face mask as often as possible when I meet other people | 1 (strongly disagree)  -  5 (strongly agree) |
| --- | --- |

*Effectiveness of lockdowns*

| Lockdowns are very effective in stopping the spread of COVID-19 | 1 (strongly disagree)  -  5 (strongly agree) |
| --- | --- |
| So many people are affected by COVID-19 that lockdowns reduce the risk of infection | 1 (strongly disagree)  -  5 (strongly agree) |
| COVID-19 is highly contagious, so lockdown is useful | 1 (strongly disagree)  -  5 (strongly agree) |
| I don’t understand why people have ignored lockdown restrictions | 1 (strongly disagree)  -  5 (strongly agree) |

Vaccination status

Have you received at least one dose of a vaccine for COVID-19?

- Yes
- No

**(Yes)** How soon did you receive a vaccine when it was made available to you?

4. As soon as it was made available

3. Less than a month after it was made available

2. 1-2 months after it was made available

1. 3+ months after it was made available

**(No)** Will you get vaccinated against COVID-19 when it is made available to you?

1. I will definitely get vaccinated
2. I will probably get vaccinated
3. I may or may not get vaccinated
4. I will probably *not* get vaccinated
5. I will definitely *not* get vaccinated

Pandemic Fatigue Scale (PFS)

“Please indicate the extent to which you disagree or agree with the following statements.”

| *Information fatigue* |  |
| --- | --- |
| I am tired of all the COVID-19 discussions in TV shows, newspapers, and radio programs, etc. | 1 (strongly disagree)  -  7 (strongly agree) |
| I am sick of hearing about COVID-19 | 1 (strongly disagree)  -  7 (strongly agree) |
| When friends or family members talk about COVID-19, I try to change the subject because I do not want to talk about it anymore | 1 (strongly disagree)  -  7 (strongly agree) |
| *Behavioural fatigue* |  |
| I feel strained from following all of the behavioural regulations and recommendations around COVID-19 | 1 (strongly disagree)  -  7 (strongly agree) |
| I am tired of restraining myself to save those who are most vulnerable to COVID-19 | 1 (strongly disagree)  -  7 (strongly agree) |
| I am losing my spirit to fight against COVID-19 | 1 (strongly disagree)  -  7 (strongly agree) |

Demographics

What is your gender?

- Male
- Female
- Non-binary / third gender
- Other: [*text entry*]
- Prefer not to say

What is your age?

- [*Drop-down box, 18-100*]
- Prefer not to say

What is your nationality?

- Nationality 1: [*text entry*]
- Nationality 2: [*text entry*]
- Nationality 3: [*text entry*]
- Prefer not to say

In what country have you lived in the longest? Please write your answer in the box provided below.

- [*text entry*]
- Prefer not to say

Have you been living in Australia since March 20, 2020?

- Yes
- No

What state or territory to live in? (drop-down box)

- New South Wales
- Victoria
- Queensland
- Western Australia
- South Australia
- Tasmania
- Australian Capital Territory
- Northern Territory

(NSW) What city or town do you live in?

- Sydney
- Newcastle
- Central Coast
- Wollongong
- Maitland
- Tweed Heads
- Wagga Wagga
- Albury
- Coffs Harbour
- Port Macquarie
- Orange
- Queanbeyan
- Dubbo
- Tamworth
- Other: [*text entry*]

(If Sydney was selected) What local government area (LGA) of Sydney do you live in?

- [*list of 27 LGAs in Sydney listed on NSW Health website*]

(Victoria) What city or town do you live in?

- Melbourne
- Geelong
- Ballarat
- Bendigo
- Melton
- Mildura
- Shepparton
- Wodonga
- Sunbury
- Warrnambool
- Traralgon
- Wangaratta
- Other: [*text entry*]

(Queensland) What city or town do you live in?

- Brisbane
- Gold Coast
- Sunshine Coast
- Townsville
- Cairns
- Toowoomba
- Mackay
- Rockhampton
- Bundaberg
- Maryborough
- Mount Isa
- Gympie
- Other: [*text entry*]

(Western Australia) What city or town do you live in?

- Perth
- Bunbury
- Geraldton
- Kalgoorlie-Boulder
- Albany
- Busselton
- Karratha
- Port Hedland
- Esperance
- Other: [*text entry*]

(South Australia) What city or town do you live in?

- Adelaide
- Gawler
- Mount Gambier
- Whyalla
- Murray Bridge
- Victor Harbor
- Crafers-Bridgewater
- Port Pirie
- Port Lincoln
- Port Augusta
- Other: [*text entry*]

(Tasmania) What city or town do you live in?

- Hobart
- Launceston
- Devonport
- Burnie-Somerset
- Ulverstone
- Other: [*text entry*]

(ACT) What city or town do you live in?

- Canberra
- Other: [*text entry*]

(Northern Territory) What city or town do you live in?

- Darwin
- Palmerston
- Alice Springs
- Litchfield
- Katherine
- Other: [*text entry*]

Attention check

| This is an attention check. Please click "4". | 1 (strongly disagree)  -  5 (strongly agree) |
| --- | --- |

Miscellaneous methods

For Study 1, participants completed the study in exchange for £0.80. Pounds were used for the currency because they were the default currency for the Prolific platform. Study 2 excluded people who completed Study 1. Participants completed the study in exchange for £1.25, accounting for the extra time needed to complete Study 2 compared to Study 1.

References to ‘coronavirus’ in the Pfattheicher et al.^1,2^ state empathy and vaccination measures was replaced with COVID-19 to conform with WHO naming conventions at the time of the study^3^.

During the experimental manipulation section, the ‘next’ button to proceed with the study was hidden and displayed only after 60 second has passed for both experimental conditions.

Sample characteristics

Study 1 total participants: 299

Study 2 total participants: 301

Vaccination status: 467 yes, 133 no

Residence: 198 Victoria, 190 NSW, 95 Queensland, 53 Western Australia, 39 South Australia, 14 ACT, 10 Tasmania, 1 Northern Territory

NSW Residence: 72 Sydney, 21 not Sydney

Sydney Residence: 39 hard lockdown, 33 lockdown

Nationality: 466 Australian, 21 British, 15 Indian, 14 Chinese, 10 Vietnamese, 9 New Zealander, 9 Malaysian, 8 Indonesian, 34 other, 15 Prefer not to say

Country lived in longest: 477 Australia, 13 United Kingdom, 11 India, 8 Indonesia, 7 New Zealand, 6 Malaysia, 74 Other, 9 Prefer not to say

*Key: NSW, Victoria and ACT were in lockdown at time of studies*

*Select local government areas in Sydney, NSW were under hard lockdown compared to other LGAs when Study 1 was conducted*

Texts used in Study 2

Experimental condition

*Modified to preserve subject anonymity*

In the following, we ask you to read a story (source: The Guardian):

Person 1 believed that if it wasn’t for their sibling Person 2, they wouldn’t exist. When seven year-old Person 2 realized that their parents weren’t going to live forever, they asked for a sibling so they would never be alone.

By the spring of 2020, at ages 21 and 29, Person 1 and Person 2 shared a condo in Anaheim,

California, not far from Disneyland, which they both loved.

Both worked at a 147-bed locked nursing facility that specialized in caring for elderly people

with cognitive issues such as Alzheimer’s, where Person 1, a nursing student, was mentored by Person 2, a registered nurse.

Both got tested for Covid-19 on the same day in June.

Both tests came back positive.

Yet only one of them survived.

Less than two months before Person 2 and Person 1 fell ill, Person 2 posted two pictures to

Instagram: one was a photo of a firework display at Disneyland, the other was a picture of

themselves in medical scrubs, wearing a face mask, giving the peace sign.

“Heeeeeyo! It’s been a minute,” they wrote in the caption. “It’s been a tough month for all of us.” They worked with a vulnerable population, they said, and “it’s just mentally exhausting thinking each night when I come home that I may be having symptoms the next day.”

Even so, Person 2 was the kind of helpful, empathetic nurse who “makes things easier for everybody”, said colleague Person 3. They knew how to talk to patients and was attuned to others’ stress levels. “We were so busy, and it was, ‘I’ll buy you lunch, I’ll buy you dinner, I’ll buy you boba.’”

It had been about 35 days since Disneyland closed its gates, Person 2 noted in their post. Person 2’s photos – of the Sleeping Beauty castle framed by tabebuia blossoms, or of themself in an attention grabbing Little Mermaid sweater – and corny jokes endeared them to thousands of followers on Instagram. “[They] had a way of capturing magic,” said their friend Person 4. The pictures were joyful, like memories of childhood.

Person 2’s last post was on 10 June, announcing that Disneyland planned to reopen in July. At some point the virus had reached their nursing home, infecting 49 staff and 120 residents and ultimately killing 14 people.

Control condition (source: <https://en.wikipedia.org/wiki/Sugarcane>)

In the following, we ask you to read the following (source: Wikipedia):

Sugarcane or sugar cane refers to several species and hybrids of tall perennial grass in the genus Saccharum, tribe Andropogoneae, that are used for sugar production. The plants are 2–6 m (6–20 ft) tall with stout, jointed, fibrous stalks that are rich in sucrose, which accumulates in the stalk internodes. Sugarcanes belong to the grass family, Poaceae, an economically important flowering plant family that includes maize, wheat, rice, and sorghum, and many forage crops. It is native to the warm, temperate tropical regions of India, Southeast Asia, and New Guinea. The plant is also grown for biofuel production, especially in Brazil, as the canes can be used directly to produce ethyl alcohol (ethanol).

Grown in tropical and subtropical regions, sugarcane is the world's largest crop by production quantity, with 1.8 billion Tonnes produced in 2017, with Brazil accounting for 40% of the world total. In 2012, the Food and Agriculture Organization estimated it was cultivated on about 26×106 ha (64×106 acres), in more than 90 countries. Sugarcane accounts for 79% of sugar produced globally (most of the rest is made from sugar beets). About 70% of the sugar produced comes from Saccharum officinarum and its hybrids. All sugarcane species can interbreed, and the major commercial cultivars are complex hybrids.

Sucrose (table sugar) is extracted from sugarcane in specialized mill factories. It is consumed directly in confectionery, used to sweeten beverages, as a preservative in jams and conserves, as a decorative finish for cakes and pâtisserie, as a raw material in the food industry, or fermented to produce ethanol. Products derived from fermentation of sugar include falernum, rum, and cachaça. In some regions, people use sugarcane reeds to make pens, mats, screens, and thatch. The young, unexpanded flower head of Saccharum edule (duruka) is eaten raw, steamed, or toasted, and prepared in various ways in Southeast Asia, including Fiji and certain island communities of Indonesia.

Sugarcane was an ancient crop of the Austronesian and Papuan people. It was introduced to Polynesia, Island Melanesia, and Madagascar in prehistoric times via Austronesian sailors. It was also introduced to southern China and India by Austronesian traders around 1200 to 1000 BC. The Persians and Greeks encountered the famous "reeds that produce honey without bees" in India between the sixth and fourth centuries BC. They adopted and then spread sugarcane agriculture.

Study 1 and 2 analyses

Prior to dataset combination, analyses were conducted separately on the Study 1 and Study 2 datasets.

Study 1

A series of Pearson and point-biserial correlation analyses were run on the Study 1 dataset (*n* = 299) to test the prediction that there would be positive associations between empathy, support for public health measures, and vaccination intention alongside negative relationships between pandemic fatigue and empathy, support for public health measures, and vaccination intention (Table S1). As predicted, there was a strong positive association between empathy and support for public health measures (*r*[297] = .518, *p* < .001), but there was no association between empathy and vaccination status (*p* = .091), or empathy and vaccination willingness (*p* = .187). Vaccination ratings were used to compute a vaccination willingness index. There was no relationship between support for public health measures and vaccination status (*p* = .057), but there was a weak positive relationship between support for public health measures and vaccination willingness (*r*[297] = .168, *p* = .004)].

As predicted, there was a moderate negative relationship between pandemic fatigue and empathy (*r*[297] = -.251, *p* < .001), and there was a moderate negative association between pandemic fatigue and support for public health measures (*r*[297] = -.392, *p* < .001). However, there was no relationship between pandemic fatigue and vaccination status (*p* = .253) or willingness to get vaccinated (*p* = .474). Additional analyses found a weak positive relationship between support public health measures and being in a lockdown (*r*[297] = .129, *p* = .026), and a moderate positive association between being under lockdown and pandemic fatigue (*r*[297] = .257, *p* < .001).

Correlational analyses for Study 1 at the sample size needed to find an effect found strong associations between empathy and support for public health measures, moderate negative associations between pandemic fatigue, empathy and support for public health measures.

**Table S1.** Correlation matrix of age, empathy, support for public health measures, pandemic fatigue, vaccination status (yes/no), vaccination willingness and lockdown status (no lockdown, lockdown) for Study 1. Pearson’s *r*, with 95% confidence intervals shown in brackets. * *p* < .05, ** *p* < .01, *** *p* < .001. ^1^= point biserial correlation was calculated.

|  | Age | Empathy | Public health | Pandemic fatigue | Vaccination status | Vaccination willingness | Lockdown |
| --- | --- | --- | --- | --- | --- | --- | --- |
| Age | – |  |  |  |  |  |  |
| Empathy | -.087  [-.199, .026] | – |  |  |  |  |  |
| Public health | -.21***  [-.316, -.099] | .518***  [.43, .596] | – |  |  |  |  |
| Pandemic fatigue | -.014  [-.127, .10] | -.251***  [-.354, -.141] | -.392***  [-.484, -.291] | – |  |  |  |
| Vaccination status^1^ | .03  [-.083, .143] | .098  [-.016, .209] | .11  [-.003, .221] | -.066  [-.178, .047] | – |  |  |
| Vaccination willingness | -.012  [-.126, .102] | .077  [-.037, .189] | .168**  [.055, .276] | .042  [-.073, .155] | -.164*  [-.273, -.051] | – |  |
| Lockdown^1^ | -.108  [-.219, .005] | .102  [-.012, .213] | .129**  [.016, .239] | .257***  [.148, .36] | .11  [-.004, .22] | .08  [-.034, .192] | – |

Study 2

To assess the robustness of the Study 2 dataset, we ran separate hierarchical multiple regressions for support for public health measures and vaccination status. A moderated hierarchical linear regression investigating support for public health measures as the outcome variable was conducted by assessing condition (empathy manipulation: experimental vs control), pandemic fatigue, empathy, and empathy × pandemic fatigue interaction as predictors (see Table S2). Model 1 including just the empathy manipulation was not significant (*p* = .193), while Model 2 incorporating pandemic fatigue was significant and explained 13.8% of the total model variance, with pandemic fatigue decreasing support for public health measures (*p* < .001) and condition remaining non-significant (*p* = .487). Including empathy in the third model explained an additional 21.0% of the model variance (*p* < .001), with manipulation remaining non-significant (*p* = .827), pandemic fatigue reducing (*p* < .001) and empathy increasing support for public health measures (*p* < .001). Model 4, which included the empathy × pandemic fatigue interaction, accounted for an additional 2.6% of the variance, with empathy manipulation having no effect (*p* = .887), pandemic fatigue reducing support for public health measures (*p* < .001), empathy × pandemic fatigue increasing support for public health measures (*p* < .001), and empathy no longer contributing to support for public health measures (*p* = .821). Comparing the models’ AICs indicated that Model 4 was the best predictive model.

A hierarchical binomial logistic regression was then conducted to determine the sequential effect of condition, pandemic fatigue, state empathy and empathy × pandemic fatigue interaction on the likelihood that participants were vaccinated against COVID-19 (see Table S3). Model 1 factoring in condition was not significant (*p* = .542), while Model 2 incorporating pandemic fatigue was significant (*p* = .006), accounting for 4.1% of the total variance, with pandemic fatigue decreasing the likelihood of being vaccinated (*p* = .006) and condition having no effect (*p* = .364). In Model 3, including empathy was significant (*p* = .021), accounting for 6.7% of the total variance, with empathy increasing (*p* = .021) and pandemic fatigue decreasing (*p* = .019) vaccination probability and condition remaining non-significant (*p* = .255). Model 4 incorporating empathy × pandemic fatigue was not significantly different from Model 3 (*p* = .813). Assessing the AICs showed that Model 3, which included state empathy and pandemic fatigue separately, was the most predictive model.

Thus, the results of the hierarchical multiple regression analyses for Study 2 demonstrated the failure of empathy manipulation to modulate support for public health measures or likelihood of receiving a COVID-19 vaccine, and matched that of the combined dataset use for the main analyses.

**Table S2***.* Hierarchical linear regression of support for public health measures predicted by condition, pandemic fatigue, empathy, and empathy × pandemic fatigue interaction for Study 2. Condition reference level set to ‘control’ (vs. empathy). Abbreviations: PF (pandemic fatigue), AIC (Akaike information criterion). * *p* < .05, ** *p* < .01, *** *p* < .001.

|  |  | 95% Confidence Interval | |  | |  |  |  |
| --- | --- | --- | --- | --- | --- | --- | --- | --- |
| **Variable** | **β** | **Lower** | **Upper** | ***t*** | | ***p*** | **Δ*R^2^*** | **AIC** |
| Block 1 |  |  |  | |  |  | .006 | 675.591 |
| Intercept |  |  |  | 70.381 | | <.001*** |  |  |
| Condition | .15 | [-.076, | .377] | 1.306 | | .193 |  |  |
| Block 2 |  |  |  |  | |  | .138 | 632.453 |
| Intercept |  |  |  | 40.446 | | <.001*** |  |  |
| Condition | .075 | [-.137, | .287] | 0.696 | | .487 |  |  |
| PF | -.374 | [-.48, | -.268] | -6.944 | | <.001*** |  |  |
| Block 3 |  |  |  |  | |  | .21 | 549.264 |
| Intercept |  |  |  | 11.174 | | <.001*** |  |  |
| Condition | -.021 | [-.206, | .165] | -0.218 | | <.001*** |  |  |
| PF | -.307 | [-.40, | -.213] | -6.475 | | .827 |  |  |
| Empathy | .466 | [.373, | .56] | 9.824 | | <.001*** |  |  |
| Block 4 |  |  |  |  | |  | .026 | 539.254 |
| Intercept |  |  |  | 7.813 | | <.001*** |  |  |
| Condition | -.013 | [-.195, | .169] | -0.142 | | .887 |  |  |
| PF | -.301 | [-.392, | -.209] | -4.713 | | <.001*** |  |  |
| Empathy | .409 | [.312, | .506] | 0.227 | | .821 |  |  |
| Empathy×PF | .141 | [.063, | .22] | 3.543 | | <.001*** |  |  |

**Table S3.** Hierarchical binomial logistic regression of vaccination status predicted by condition, pandemic fatigue, empathy, and empathy × pandemic fatigue interaction for Study 2. The outcome variable is the probability of being vaccinated against COVID-19. Condition reference level set to ‘control’ (vs. empathy). Abbreviations: OR (odds ratio), PF (pandemic fatigue), AIC (Akaike information criterion), *R_N_^2^* (Nagelkerke’s *R^2^*). * *p* < .05, ** *p* < .01, *** *p* < .001.

|  |  | 95% Confidence Interval | |  |  |  |  |
| --- | --- | --- | --- | --- | --- | --- | --- |
| **Variables** | **OR** | **Lower** | **Upper** | ***Z*** | ***p*** | **Δ*R_N_^2^* (χ^2^_Sig_)** | **AIC** |
| Block 1 |  |  |  |  |  | .002 (.542) | 317.709 |
| Intercept |  |  |  | 6.745 | <.001*** |  |  |
| Condition | 0.843 | [.486, | 1.461] | -0.609 | .542 |  |  |
| Block 2 |  |  |  |  |  | .039 (.006**) | 312.009 |
| Intercept |  |  |  | 5.19 | <.001*** |  |  |
| Condition | 0.771 | [.44, | 1.352] | -0.907 | .364 |  |  |
| PF | 0.755 | [.617, | .924] | -2.723 | .006** |  |  |
| Block 3 |  |  |  |  |  | .026 (.021*) | 308.675 |
| Intercept |  |  |  | 0.58 | .562 |  |  |
| Condition | 0.717 | [.405, | 1.271] | -1.139 | .252 |  |  |
| PF | 0.781 | [.636, | .959] | -2.355 | .019* |  |  |
| Empathy | 1.539 | [1.068, | 2.22] | 2.311 | .021* |  |  |
| Block 4 |  |  |  |  |  | .000 (.813) | 310.619 |
| Intercept |  |  |  | 0.008 | .994 |  |  |
| Condition | 0.716 | [.404, | 1.269] | -1.143 | .253 |  |  |
| PF | 0.879 | [.326, | 2.371] | -0.256 | .798 |  |  |
| Empathy | 1.747 | [.575, | 5.31] | 0.984 | .325 |  |  |
| Empathy×PF | 0.973 | [.774, | 1.222] | -0.237 | .813 |  |  |

Exploratory analyses

All exploratory analyses were not pre-registered.

Additional analyses were run after separating pandemic fatigue into the two factors of information fatigue and behavioural fatigue of the Pandemic Fatigue Scale^4^. Correlations were run using information fatigue and behavioural fatigue as separate variables (Table S4). Information fatigue was negatively correlated with empathy (*r*[598] = -.182, *p* < .001), support for public health measures (*r*[598] = -.333, *p* < .001), and vaccination status (*r*[598] = -.125, *p* = .002), and positively correlated with lockdown status (*r*[598] = .153, *p* < .001). There were no associations between information fatigue and vaccination willingness (*p* = .461). Behavioural fatigue was negatively correlated with empathy (*r*[598] = -.186, *p* < .001), support for public health measures (*r*[598] = -.361, *p* < .001), and vaccination willingness (*r*[598] = -.092, *p =* .025), while being positively correlated with lockdown status (*r*[598] = .309, *p* < .001) and not correlated at all with vaccination status (*p* = .08). Unsurprisingly, there was a strong positive correlation between information fatigue and behavioural fatigue (*r*[598] = .625, *p* < .001).

A chi-square test of independence was run to examine the relationship between vaccination status and lockdown status. This showed a significant association between these variables (χ^2^[1, *N* = 600] = 14.329, *p* < .001), indicating that participants under lockdown restrictions were more likely to be vaccinated than participants not in lockdown.

**Table S4.** Correlation matrix of empathy, support for public health measures, vaccination status (yes/no), vaccination willingness, lockdown status (no lockdown, lockdown), information fatigue, and behavioural fatigue. Pearson’s *r*, with 95% confidence intervals shown in brackets. * *p* < .05, ** *p* < .01, *** *p* < .001. ^1^= point biserial correlation was calculated.

|  | Empathy | Public health | Vaccination status | Vaccination willingness | Lockdown | Information fatigue | Behavioural fatigue |
| --- | --- | --- | --- | --- | --- | --- | --- |
| Empathy | – |  |  |  |  |  |  |
| Public health | .515***  [.453, .571] | – |  |  |  |  |  |
| Vaccination status^1^ | .128**  [.048, .206] | .172***  [.093, .248] | – |  |  |  |  |
| Vaccination willingness | .106**  [.026, .185] | .201**  [.123, .277] | -.10*  [-.179, -.02] | – |  |  |  |
| Lockdown^1^ | .089*  [.009, .168] | .123**  [.043, .201] | .155***  [.075, .232] | .005  [-.075, .085] | – |  |  |
| Information fatigue | -.182***  [-.259, -.104] | -.333***  [-.402, -.26] | -.125**  [-.203, -.045] | -.03  [-.146, .014] | .153***  [.074, .23] | – |  |
| Behavioural fatigue | -.186***  [-.262, -.107] | -.361***  [-.429, -.289] | -.071  [-.151, .009] | -.092*  [-.171, -.012] | .309***  [.234, .379] | .625***  [.574, .671] | – |

Two two-tailed Mann-Whitney *U* tests with a Benjamini-Hochberg procedure applied to keep the false discovery rate controlled at .05 were conducted assessing the effect of vaccination status on information fatigue and behavioural fatigue. There was a significant difference in information fatigue between the vaccinated (*Mdn* = 4.0, *M* = 4.082, *SD* = 1.665) and unvaccinated groups (*Mdn* = 4.667, *M* = 4.579, *SD* = 1.549; *U*[*N*_vacc_ = 467, *N*_unvacc_ = 133] = 25809, *p* = .003, *r*_rb_ = .169), suggesting that vaccinated participants experienced less information fatigue than unvaccinated participants. There was no difference in behavioural fatigue between vaccinated and unvaccinated participants (*p* = .090). These indicate that in the domain of pandemic fatigue, vaccinated people specifically felt less information fatigue than unvaccinated people but that there was no effect for behavioural fatigue. It should, however, be stressed that we cannot infer causation from these results, and it may be possible that vaccinated people were less susceptible to information fatigue due to some unaccounted third factor.

Another hierarchical linear regression was run assessing information fatigue, behavioural fatigue, empathy, empathy × information fatigue, empathy × behavioural fatigue, gender, lockdown status, age, and age × empathy as predictors and support for public health measures as the outcome variable (Table S2). Information fatigue accounted for 10.9% of the total model variance (*p* < .001). Including behavioural fatigue into the second block explained an additional 3.9% of the model variance, with information fatigue (*p* < .001) and behavioural fatigue (*p* < .001) predicting reduced support for public health measures. Including empathy in the third block explained an additional 19.7% of the variance in the model, with empathy (*p* < .001) contributing to support for public health measures, and information fatigue (*p* = .003) and behavioural fatigue reducing support for public health measures (*p* < .001). Adding empathy × information fatigue and empathy × behavioural fatigue to the model added an additional 3.2% of the variance, with empathy × information fatigue (*p* = .024) increasing support for public health measures, information fatigue (*p* = .008) and behavioural fatigue (*p* = .015) reducing support for public health measures, empathy × behavioural fatigue having no effect (*p* = .076), and empathy no longer contributing to support for public health measures (*p* = .769). Adding gender to the model explained an additional 1.7% of the variance, with information fatigue (*p* = .004), behavioural fatigue (*p* = .009) and being male (*p* < .001) reducing support for public health measures, empathy × behavioural fatigue having no effect (*p* = .053), and empathy × information fatigue increasing support for public health measures (*p* = .014).

Adding lockdown status, age and age × empathy to the model explained an additional 4.9% of the model variance, with empathy (*p* = .005), empathy × information fatigue (*p* = .017), empathy × behavioural fatigue (*p* = .031), being male (*p* < .001), being under lockdown (*p* < .001), and age × empathy (*p* = .004) associated with increased support for public health measures, and information fatigue (*p* = .011), behavioural fatigue (*p* = .001), and age (*p* = .001) associated with reduced support for public health measures. Assessing the model AICs revealed that Model 6 was the most predictive model.

**Table S5**. Hierarchical linear regression of support for public health measures predicted by information fatigue, behavioural fatigue, empathy, empathy × information fatigue, empathy × behavioural fatigue, gender, lockdown status, age and age × empathy. Reference level for gender was set as ‘female’ and for lockdown was set as ‘no lockdown’. Abbreviations: IF (information fatigue), BF (behavioural fatigue). * *p* < .05, ** *p* < .01, *** *p* < .001.

|  |  | 95% Confidence Interval | |  |  |  |  |
| --- | --- | --- | --- | --- | --- | --- | --- |
| **Variable** | **β** | **Lower** | **Upper** | ***t*** | ***p*** | **Δ*R^2^*** | **AIC** |
| Block 1 |  |  |  |  |  | .109 | 1228.068 |
| Intercept |  |  |  | 64.987 | <.001*** |  |  |
| IF | -.33 | [-.406, | -.254] | -8.522 | <.001*** |  |  |
| Block 2 |  |  |  |  |  | .039 | 1203.332 |
| Intercept |  |  |  | 64.53 | <.001*** |  |  |
| IF | -.172 | [-.267, | -.077] | -3.555 | <.001*** |  |  |
| BF | -253 | [-.348, | -.158] | -5.216 | <.001*** |  |  |
| Block 3 |  |  |  |  |  | .197 | 1048.366 |
| Intercept |  |  |  | 16.334 | <.001*** |  |  |
| IF | -.126 | [-.21, | -.043] | -2.965 | .003** |  |  |
| BF | -.199 | [-.283, | -.115] | -4.655 | <.001*** |  |  |
| Empathy | .453 | [.386, | .519] | 13.354 | <.001*** |  |  |
| Block 4 |  |  |  |  |  | .032 | 1022.412 |
| Intercept |  |  |  | 11.625 | <.001*** |  |  |
| IF | -.138 | [-.221, | -.056] | -2.663 | .008** |  |  |
| BF | -.178 | [-.26, | -.095] | -2.436 | .015* |  |  |
| Empathy | .405 | [.337, | .472] | -0.293 | .769 |  |  |
| Empathy×IF | .103 | [.013, | .193] | 2.256 | .024* |  |  |
| Empathy×BF | .074 | [-.008, | .155] | 1.779 | .074 |  |  |
| Block 5 |  |  |  |  |  | .017 | 1009.469 |
| Intercept |  |  |  | 12.336 | <.001*** |  |  |
| IF | -.141 | [-.222, | -.059] | -2.874 | .004** |  |  |
| BF | -.185 | [-.266, | -.103] | -2.626 | .009** |  |  |
| Empathy | .376 | [.308, | .445] | -0.95 | .343 |  |  |
| Empathy×IF | .111 | [.022, | .20] | 2.46 | .014* |  |  |
| Empathy×BF | .08 | [-.001, | .16] | 1.939 | .053 |  |  |
| Gender |  |  |  |  |  |  |  |
| *Male* | -.242 | [-.375, | -.109] | -3.572 | <.001*** |  |  |
| *Non-binary* | -.081 | [-.081, | .979] | 1.663 | .097 |  |  |
| Block 6 |  |  |  |  |  | .049 | 964.806 |
| Intercept |  |  |  | 11.666 | <.001*** |  |  |
| IF | -.124 | [-.203, | -.046] | -2.539 | .011* |  |  |
| BF | -.263 | [-.345, | -.181] | -3.412 | <.001*** |  |  |
| Empathy | .324 | [.256, | .393] | -2.841 | .005** |  |  |
| Empathy×IF | .094 | [.008, | .18] | 2.157 | .031* |  |  |
| Empathy×BF | .095 | [.017, | .173] | 2.399 | .017* |  |  |
| Gender |  |  |  |  |  |  |  |
| *Male* | -.228 | [-.358, | -.098] | -3.442 | <.001*** |  |  |
| *Non-binary* | .384 | [-.126, | .894] | 1.479 | .14 |  |  |
| Lockdown | .365 | [.226, | .504] | 5.161 | <.001*** |  |  |
| Age | -.104 | [-.166, | -.042] | -3.387 | <.001*** |  |  |
| Age×Empathy | .094 | [.031, | .157] | 2.921 | .004** |  |  |

A hierarchical binomial logistic regression analysis was conducted to ascertain the effects of information fatigue, behavioural fatigue, empathy, empathy × information fatigue, empathy × behavioural fatigue, gender, and lockdown status on the likelihood that participants are vaccinated (Table S3). The first block of the logistic regression model with information fatigue was significant (*p* = .004), but the second model incorporating behavioural fatigue was not significant (*p* = .840). Adding empathy to the model accounted for an additional 1.4% of the model variance, where empathy was associated with an increased likelihood of being vaccinated (*p* = .016) but information fatigue associated with reduced likelihood of being vaccinated (*p* = .025). Adding empathy × information fatigue and empathy × behavioural fatigue to the model did not contribute to the model (*p* = .624). The fifth block adding gender was not statistically significant (*p* = .121). When lockdown status was added, the model change was statistically significant (*p* < .001). Locked-down participants were 2.557 times more likely to be vaccinated than no-lockdown participants and no other variables contributed to the likelihood that participants were vaccinated (*p*’s > .057). Assessing the model AICs revealed that Model 6 was the most predictive model.

**Table S6.** Hierarchical binomial logistic regression of vaccination status predicted by condition, state empathy, support for public health measures, lockdown status, information fatigue, behavioural fatigue and gender in Study 2. Reference level for condition was set as ‘control’, lockdown status set as ‘no lockdown’ and gender was set as ‘female’. Abbreviations: IF (information fatigue), BF (behavioural fatigue), *R_N_^2^* (Nagelkerke’s *R^2^*). * *p* < .05, ** *p* < .01, *** *p* < .001.

|  |  | 95% Confidence Interval | |  |  |  | |  | |  | |
| --- | --- | --- | --- | --- | --- | --- | --- | --- | --- | --- | --- |
| **Variables** | **Odds** | **Lower** | **Upper** | ***Z*** | ***p*** | | **Δ*R_N_^2^* (χ^2^_Sig_)** | | **AIC** | |  |
| Block 1 |  |  |  |  |  | | .022 (.003**) | | 626.179 | |  |
| Intercept | 7.63 | [4.301, | 13.534] | 6.949 | <.001*** | |  | |  | |  |
| IF | 0.836 | [.741, | 0.944] | -2.896 | .004** | |  | |  | |  |
| Block 2 |  |  |  |  |  | | .0 (.839) | | 628.137 | |  |
| Intercept | 7.49 | [4.112, | 13.643] | 6.582 | <.001*** | |  | |  | |  |
| IF | 0.828 | [0.711, | 0.965] | -2.423 | .015* | |  | |  | |  |
| BF | 1.017 | [0.866, | 1.193] | 0.204 | .839 | |  | |  | |  |
| Block 3 |  |  |  |  |  | | .014 (.017*) | | 624.428 | |  |
| Intercept | 1.653 | [0.427, | 6.399] | 0.727 | .467 | |  | |  | |  |
| IF | 0.839 | [0.719, | 0.978] | -2.236 | .025* | |  | |  | |  |
| BF | 1.039 | [0.883, | 1.222] | 0.461 | .461 | |  | |  | |  |
| Empathy | 1.375 | [1.062, | 1.78] | 2.42 | .016* | |  | |  | |  |
| Block 4 |  |  |  |  |  | | .003 (.624) | | 627.485 | |  |
| Intercept | 0.455 | [0.015, | 14.022] | -0.45 | .652 | |  | |  | |  |
| IF | 1.334 | [0.497, | 3.585] | 0.572 | .567 | |  | |  | |  |
| BF | 0.816 | [0.324, | 2.058] | -0.431 | .667 | |  | |  | |  |
| Empathy | 1.848 | [0.848, | 4.027] | 1.546 | .122 | |  | |  | |  |
| Empathy×IF | 0.899 | [0.722, | 1.124] | -0.933 | .351 | |  | |  | |  |
| Empathy×BF | 1.056 | [0.855, | 1.305] | 0.504 | .615 | |  | |  | |  |
| Block 5 |  |  |  |  |  | | .01 (.121) | | 627.267 | |  |
| Intercept | 1.006 | [0.029, | 35.128] | 0.003 | .997 | |  | |  | |  |
| IF | 1.278 | [0.475, | 3.44] | 0.486 | .627 | |  | |  | |  |
| BF | 0.765 | [0.298, | 1.961] | -.559 | .576 | |  | |  | |  |
| Empathy | 1.603 | [0.72, | 3.572] | 1.155 | .248 | |  | |  | |  |
| Empathy×IF | 0.908 | [0.726, | 1.135] | -0.846 | 398 | |  | |  | |  |
| Empathy×BF | 1.07 | [0.863, | 1.328] | 0.618 | .536 | |  | |  | |  |
| Gender |  |  |  |  |  | |  | |  | |  |
| *Male* | 0.691 | [0.46, | 1.037] | -1.783 | .075 | |  | |  | |  |
| *Non-binary* | 2.355 | [0.252, | 21.965] | 0.752 | .452 | |  | |  | |  |
| Block 6 |  |  |  |  |  | | .043 (<.001***) | | 612.031 | |  |
| Intercept | 1.11 | [0.031, | 40.341] | 0.057 | .955 | |  | |  | |  |
| IF | 1.272 | [0.47, | 3.445] | 0.474 | .636 | |  | |  | |  |
| BF | 0.718 | [0.279, | 1.844] | -0.689 | .491 | |  | |  | |  |
| Empathy | 1.501 | [0.667, | 3.38] | 0.981 | .327 | |  | |  | |  |
| Empathy×IF | 0.911 | [0.728, | 1.141] | -0.809 | .418 | |  | |  | |  |
| Empathy×BF | 1.057 | [0.852, | 1.311] | 0.501 | .617 | |  | |  | |  |
| Gender |  |  |  |  |  | |  | |  | |  |
| *Male* | 0.669 | [0.442, | 1.013] | -1.901 | .057 | |  | |  | |  |
| *Non-binary* | 1.789 | [0.195, | 16.38] | 0.515 | .607 | |  | |  | |  |
| Lockdown | 2.557 | [1.638, | 3.992] | 4.131 | <.001*** | |  | |  | |  |

COVID-19 pandemic in Australia

Australia is a federation divided into six states and two self-governing territories, each with broad remit to implement public health orders independently of one another and the federal government. On March 20, 2020, the federal government closed the international border to non-citizens and introduced 14-day mandatory quarantine in government-designated facilities, such as hotels or dedicated quarantine facilities. This was followed by Australia entering a national lockdown from March 23^5^. Although the federal government opted for a suppression strategy for COVID-19 in agreement with state leaders, the states and territories adhered to an unofficial elimination strategy^6–8^. This resulted in Australia recording relatively low case numbers and deaths, in addition to lower lockdown severity and less disruptions to everyday life and the economy, compared to countries that adopted a mitigation strategy^9,10^.

Due to the nature of the federation, and intermittent state border closures^11^, each state and territory had different lived experiences of the COVID-19 pandemic. For example, the Australian city of Melbourne was identified as the single most locked down city in the world^12^, being under lockdown for a total of 262 days across six lockdowns since the WHO declared COVID-19 a pandemic in March 2020^13^. In contrast, the capital cities of other states such as Queensland (Brisbane) and Western Australia (Perth) experienced repeated but brief lockdowns totalling 14-18 days in lockdown and experiencing minimal restrictions throughout 2021.

Popular support for COVID-related public health measures in Australia remained high during the pandemic. A survey by the Lowy Institute conducted in March 2021 found that 95% of Australians believe Australia handled the pandemic well (65% very well, 30% fairly well)^14^. Regarding pandemic restrictions and freedoms, a Pew Research Center survey conducted in March-May 2021 reported that 72% of Australians believed that the Australian government respected personal freedoms, and in another question found that 68% of Australians believed that COVID restrictions were “about right” with 17% wanting fewer restrictions and 14% wanting more restrictions^15^. Additionally, data collected for the Australian Survey of Social Attitudes between February-June 2021 found that 65% of Australians believed lockdowns rules were “about right”, compared to 19% who found lockdown rules “probably” or “definitely too restrictive”, and 15% who found lockdown rules “probably” or “definitely not restrictive enough”^16^.

At the time this research was conducted, the cities of Sydney (NSW), Melbourne (Victoria) and Canberra (Australian Capital Territory) were under lockdown orders, in addition to smaller towns and cities in NSW and Victoria where the virus was detected, due to a lapse in quarantine protocols in Sydney that spread throughout the city then across state borders. All regions of NSW, Victoria and the ACT exited lockdown throughout October 2021 following rapid uptake of COVID-19 vaccinations and the abandonment of the COVID-19 elimination strategy in virus-affected states^17^. To obtain a broad sample of the Australian population with different experiences of the pandemic, including self-reported support for public health measures and pandemic fatigue, we conducted our research online. As evident in the pandemic chronology, this research was conducted before the detection of the Omicron variant of COVID-19, which sparked a large wave across Australia from December 2021 to January 2022.

**References**

1. Pfattheicher, S., Nockur, L., Böhm, R., Sassenrath, C. & Petersen, M. B. The emotional path to action: Empathy promotes physical distancing and wearing of face masks during the COVID-19 pandemic. *Psychol Sci* **31**, 1363–1373 (2020).

2. Pfattheicher, S., Petersen, M. B. & Böhm, R. Information about herd immunity through vaccination and empathy promote COVID-19 vaccination intentions. *Health Psychology* (2021) doi:10.1037/hea0001096.

3. World Health Organisation [WHO]. Naming the coronavirus disease (COVID-19) and the virus that causes it. https://www.who.int/emergencies/diseases/novel-coronavirus-2019/technical-guidance/naming-the-coronavirus-disease-(covid-2019)-and-the-virus-that-causes-it (2020).

4. Lilleholt, L., Zettler, I., Betsch, C. & Böhm, R. Pandemic fatigue: Measurement, correlates, and consequences. Preprint at https://doi.org/10.31234/osf.io/2xvbr (2021).

5. Prime Minister of Australia. Update on coronavirus measures. https://www.pm.gov.au/media/update-coronavirus-measures-220320 (2020).

6. McNeill, L. P., Heather. ‘Crush and kill it’: WA Premier says COVID-19 elimination is the right approach. *WAtoday* https://www.watoday.com.au/national/western-australia/nitpicking-undermining-should-stop-wa-premier-to-keep-border-closed-to-queensland-20210110-p56t1v.html (2021).

7. Packham, C. Australia eliminates coronavirus in many areas, to ease curbs. *Reuters* (2020).

8. The Guardian. South Australia and NSW record new Covid-19 cases as Victoria passes elimination benchmark. *The Guardian* (2020).

9. Baker, M. G., Wilson, N. & Blakely, T. Elimination could be the optimal response strategy for COVID-19 and other emerging pandemic diseases. *BMJ* **371**, m4907 (2020).

10. Oliu-Barton, M. *et al.* SARS-CoV-2 elimination, not mitigation, creates best outcomes for health, the economy, and civil liberties. *The Lancet* **397**, 2234–2236 (2021).

11. SBS News. Border closures re-imposed around Australia as more coronavirus cases reported. *SBS News* https://www.sbs.com.au/news/border-closures-re-imposed-around-australia-as-more-coronavirus-cases-reported/76bc9cf7-4027-4833-a15a-86b877c59630 (2021).

12. Boaz, J. Melbourne passes Buenos Aires’ world record for time spent in lockdown. *ABC News* (2021).

13. Kelly, L. Melbourne to ease world’s longest COVID-19 lockdowns as vaccinations rise. *Reuters* (2021).

14. Lowy Institute. COVIDpoll 2021. (2021).

15. Pew Research Center. *Global Attitudes & Trends Spring 2021*. https://www.pewresearch.org/global/dataset/spring-2021-survey-data/ (2021).

16. Tranter, B. K. Your money or your life? Public support for health initiatives during the COVID-19 pandemic. *Australian Journal of Social Issues* **n/a**, 1–18 (2022).

17. Dow, A., Massola, J. & Smethurst, A. Victoria ‘unlikely’ to eliminate Delta as PM backs NSW on reopening. *The Sydney Morning Herald* https://www.smh.com.au/politics/federal/victoria-unlikely-to-eliminate-delta-as-pm-backs-nsw-on-reopening-20210821-p58krm.html (2021).
